# Sedatives and analgesics are major contributors to potentially inappropriate duplicate prescriptions in geriatric psychiatry*

**DOI:** 10.1111/psyg.12940

**Published:** 2023-01-31

**Authors:** Martin Schulze Westhoff, Sebastian Schröder, Adrian Groh, Helge Frieling, Stefan Bleich, Felix Koop, Dirk O. Stichtenoth, Benjamin Krichevsky, Johannes Heck

**Affiliations:** ^1^ Department of Psychiatry, Social Psychiatry and Psychotherapy Hannover Medical School Hannover Germany; ^2^ Institute for Clinical Pharmacology Hannover Medical School Hannover Germany; ^3^ Institute for General Practice and Palliative Care Hannover Medical School Hannover Germany; ^4^ Medical Service of the German Armed Forces Kiel Germany

**Keywords:** duplicate prescriptions, geriatric psychiatry, medication review, pharmacotherapy safety

## Abstract

**Background:**

This study sought to investigate the frequency and characteristics of duplicate prescriptions (DPs) in elderly psychiatric inpatients using a novel categorisation of DPs that differentiates between appropriate duplicate prescriptions (ADPs) and potentially inappropriate duplicate prescriptions (PIDPs).

**Methods:**

The study was conducted as a monocentric retrospective cross‐sectional pilot study on the gerontopsychiatric ward of the Department of Psychiatry, Social Psychiatry and Psychotherapy of Hannover Medical School, a large university hospital in northern Germany. The outcome measures were the nature and frequency of PIDPs compared with the frequency of ADPs.

**Results:**

For 92 individual patients a total of 339 medication chart reviews were conducted between April 2021 and February 2022. The median age of the study population was 73 years (interquartile range (IQR) 68–82 years); 64.6% were female. Patients' medications comprised a median of eight drugs (IQR 6–11 drugs) and 43.1% of the study population were exposed to at least one PIDP (at least one grade‐1 PIDP: 39.5%; at least one grade‐2 PIDP: 5.0%; at least one grade‐3 PIDP: 1.5%). Sedatives were most frequently responsible for grade‐1 and grade‐2 PIDPs, while grade‐3 PIDPs were elicited exclusively by analgesics. Nearly half of the study population (49.0%) displayed at least one ADP.

**Conclusion:**

Even though the clinical implications of PIDPs are not fully established to date, we recommend that physicians who treat elderly psychiatric patients pay special attention to PIDPs, especially PIDPs elicited by sedatives. Termination of PIDPs may prevent adverse drug reactions and save healthcare expenditures.

## INTRODUCTION

The prevention of adverse drug reactions (ADRs) represents an essential goal of rational and safe pharmacotherapy, especially in geriatric patient populations.^1^ In addition to potentially inappropriate medications (PIMs) for elderly patients, duplicate prescriptions (DPs)—among other factors—have been identified as a risk factor for the development of ADRs.^2^ Of note, DPs are defined heterogeneously in the literature (Table 1), with—for example—the description of a DP as the ‘concurrent use of two drugs of the same class to treat the same condition’ falling noticeably short.^2^ To exemplify this, the co‐administration of two drugs that belong to different pharmacological classes—such as the combination of an angiotensin‐converting enzyme inhibitor with an angiotensin‐receptor blocker—can also be considered a DP.

**Table 1:** Terminologies and definitions for duplicate prescriptions (DPs) that have been used in the scientific literature, retrieved from MEDLINE via PubMed on 03 December 2022 using the following search terms: ‘DP’, ‘double prescription’, ‘duplicate medication’, ‘duplicative medication’, ‘double medication’, ‘therapeutic duplication’. The results of the literature search are displayed in descending chronological order

Authors	Year of publication	Terminology/terminologies	Definition(s)	Differentiation between appropriate and inappropriate DPs	References
Kan *et al*.	2020	Duplicate medication/DP/duplicative prescription/therapeutic duplication	‘The practice of a patient who receives identical medications (based on the first five digits of the ATC) from an identical facility (e.g., hospital or clinic) for a period of several overlaid days (i.e., total duplicative days/total prescriptive days in a specific period)’	No	22
Zwietering *et al*.	2019	Double medication	Not further specified	No	15
Tasaka *et al*.	2018	DP	‘Duplication of a drug with similar effect’ ‘Duplication of the same drug’	No	23
Heinze *et al*.	2016	Double medication	‘The overlapping prescription of the same substance (ATC5) by two different prescribers to the same patient’ ‘Our definition of 2M excludes double prescription of the same substance (ATC5) for dose optimisation, for example, cases where a patient is prescribed two different strengths of the same drug by the same prescriber, or where a combination medicinal product (e.g., lisinopril and diuretics) is prescribed simultaneously with one of its components (e.g., lisinopril)’	No	14
Takahashi *et al*.	2016	Duplicative prescription	‘Situations in which patients received prescriptions in a main therapeutic group (ATC code 2nd level, first 3 digits) from two or more sources’	No	24
O'Mahony *et al*.	2015	Duplicate drug class prescription	‘Any duplicate drug class prescription e.g. two concurrent NSAIDs, SSRIs, loop diuretics, ACE inhibitors, anticoagulants (optimisation of monotherapy within a single drug class should be observed prior to considering a new agent)’	No	4
Freytag *et al*.	2014	DP	‘DP is assumed if at least two preparations with the same active ingredient have been taken in the last 3 months’ (translated from German with DeepL Translator (https://www.deepl.com/translator; accessed 03 December 2022)	No	18
Westerlund *et al*.	2013	Drug duplication	‘Two or more drugs containing the same active ingredient taken simultaneously’ ‘Two or more drugs from the same ATC‐group taken simultaneously’ ‘Two or more drugs with similar pharmacodynamics but from different ATC‐groups taken simultaneously’	No	25
Hsu *et al*.	2011	Potential duplicate medications	‘Based on the ATC system, potential duplicate medications can be defined as follows: Two medications are said to be possibly duplicative if they are in the same therapeutic/pharmacological class (i.e., their ATC codes have the same first four digits)’	No	26
Wetterneck *et al*.	2011	Duplicate medication order/duplicate ordering error	‘A duplicate medication order was defined as two or more orders for the same medication or for medications in the same therapeutic class, one of which was clinically redundant’ ‘Duplicate ordering errors were further categorised into three categories: (i) an identical order; (ii) the same medication (but different dose, form, frequency, or route); and (iii) a different medication of the same therapeutic class’	Yes (implicitly)	27
van Leeuwen *et al*.	2010	DP	‘The concurrent use of two drugs of the same class to treat the same condition’ ‘A DP can be both desirable (e.g. long‐ and shortacting morphine) and undesirable’	Yes	2
Kinoshita *et al*.	2008	Duplicative medication	‘A situation in which drugs of the same mechanism of action according to “Today's Therapeutic Medication 2002” were administered to one patient simultaneously for one or more days by multiple medical institutions’	No	16
Kovac *et al*.	2008	Dual use (with reference to NSAIDs)	‘Dual users were defined as patients who (i) reported currently taking a prescription NSAID for arthritis and/or joint pain and over the past month reported taking an OTC NSAID for arthritis and/or joint pain daily (1, 2, or ≥3 times per day) or a couple times per week, or (ii) reported currently taking two prescription NSAIDs for arthritis and/or joint pain, or (iii) over the past month reported taking ≥2 OTC NSAIDs for arthritis and/or joint pain daily (1, 2, or ≥3 times per day) or a couple times per week’	No	17
Riechelmann *et al*.	2007	DP/duplicate prescribing/duplicate(d) medication	‘Duplicate prescribing was considered to be present when two or more drugs from the same class were prescribed to treat the same condition (e.g., morphine and codeine prescribed as routine orders for pain) or different conditions (e.g., corticosteroids to prevent delayed nausea and as anti‐inflammatory agents)’	No	28
American Society of Health‐System Pharmacists	1996	Therapeutic duplication	Not further specified	No	29
Hanlon *et al*.	1992	Therapeutic duplication/unnecessary duplication	‘Non‐beneficial or risky prescribing of two or more drugs from the same chemical or pharmacological class’	Yes (implicitly)	30
Keys *et al*.	1992	DP	Not further specified	No	31

Abbreviation: ACE, angiotensin‐converting enzyme; ATC, anatomical therapeutic chemical; ATC5, 5th level ATC code; 2M, double medication; DM, duplicate medication; NSAID, nonsteroidal anti‐inflammatory drug; OTC, over‐the‐counter; SSRI, selective serotonin reuptake inhibitor.

It is striking that to date the concept of DPs in the scientific literature has been associated almost exclusively with detrimental effects for patients, ignoring the fact that, for example, the combined use of oral antidiabetic agents (e.g., the combination of metformin with a sodium–glucose cotransporter type 2 (SGLT2) inhibitor) in patients with type‐2 diabetes mellitus can exert meaningful therapeutic benefits.^3^ The majority of existing DP definitions, including the frequently used Screening Tool of Older Persons' Prescriptions (STOPP) criteria,^4^ do not (sufficiently) differentiate between clinically appropriate and inappropriate DPs (Table 1). To overcome the currently prevailing inconsistency and largely absent consideration of DP appropriateness of DP definitions found in the scientific literature (Table 1), Heck and colleagues proposed a new categorisation of DPs that fundamentally distinguishes between appropriate duplicate prescriptions (ADPs) and potentially inappropriate duplicate prescriptions (PIDPs).^5^ ADPs represent rational and commonly used combination treatments (often as add‐on therapy, combining treatment principles with synergistic mechanisms of action), as opposed to PIDPs. PIDPs are subdivided into three different grades; higher grades reflect an increasing degree of inappropriateness (Table 2). It must be stressed, however, that the degree of inappropriateness of PIDPs per se is not equivalent to clinical severity since it is conceivable that an ADR resulting from a grade‐1 PIDP is clinically more severe than an ADR elicited by a grade‐2 or grade‐3 PIDP.

**Table 2:** Categorisation of duplicate prescriptions (DPs) into appropriate duplicate prescriptions (ADPs) and potentially inappropriate duplicate prescriptions (PIDPs). PIDPs can be subdivided into three grades; higher grades indicate an increasing degree of inappropriateness (adopted and modified from Heck *et al.* ^5^ under a Creative Commons Attribution 4.0 International Licence). Please note that the degree of inappropriateness per se does not reflect the clinical severity of an adverse drug reaction that might arise from a PIDP

	ADPs	PIDPs		
Grade	—	1	2	3
Description	Two drugs with therapeutically desired synergistic effects (i.e., established combination or add‐on treatments)	Two drugs with overlapping or comparable pharmacodynamics	Two drugs of the same therapeutic class (i.e., targeting the same molecular structure)	Two times the same drug (exceeding the recommended maximum daily dose)
Examples	HMG‐CoA reductase inhibitor + ezetimibe	ACE inhibitor + ARB	Two different opioid analgesics (e.g., buprenorphine + hydromorphone, oxycodone + tramadol)	Hydrochlorothiazide both as single agent and as partner in an antihypertensive combination product
	Metformin + SGLT2 inhibitor	PPI + H_2_ receptor antagonist	Hydrochlorothiazide + chlorthalidone	Valsartan both as single agent and in sacubitril–valsartan
	Opioid analgesic + non‐opioid analgesic	Paracetamol + ibuprofen	Ibuprofen + diclofenac	Paracetamol both as single agent and in an acetylsalicylic acid–paracetamol–caffeine combination product
	Acetylsalicylic acid + clopidogrel	Ibuprofen + metamizole	Amlodipine + lercanidipine	Diclofenac both as single agent and in a diclofenac–misoprostol combination product
	Loop diuretic + thiazide diuretic	Doxylamine + zopiclone	Diphenhydramine + doxylamine	Codeine both as single agent and in a paracetamol–codeine combination product

Abbreviation: ACE, angiotensin‐converting enzyme; ARB, angiotensin‐receptor blocker; HMG‐CoA, hydroxymethylglutaryl coenzyme A; PPI, proton pump inhibitor; SGLT2, sodium–glucose cotransporter type 2.

Elderly patients are at increased risk of developing ADRs due to altered organ functions and age‐related changes in pharmacokinetic and pharmacodynamic characteristics.^6^ Furthermore, elderly patients are often exposed to multimorbidity‐associated polypharmacy (i.e., the concomitant intake of ≥5 different drugs) and prescription of PIMs, further increasing the likelihood of developing ADRs.^7^ Gerontopsychiatric patient populations have been identified as high‐risk groups for ADR occurrence in recent years.^8^ While the influence of inappropriate polypharmacy and PIM prescriptions has been investigated quite extensively in this context,^9^, ^10^ prescribing characteristics of DPs in gerontopsychiatric patients have not been analysed comprehensively to date.

Therefore, the present study sought to investigate the frequency and characteristics of DPs in a cohort of elderly patients treated on the gerontopsychiatric ward of a large university hospital in northern Germany on the basis of weekly medication reviews conducted by an interdisciplinary expert team. Particular emphasis was put on the differentiation between ADPs and PIDPs.

## METHODS

### Ethics approval

The Ethics Committee of Hannover Medical School approved the study (No. 10206_BO_K_2022 and Amendment). The study was carried out in full accordance with the Declaration of Helsinki (1964) and its later amendments (current version dating from 2013).

### Study design and eligibility criteria

The present study was designed as a retrospective monocentric cross‐sectional pilot study and as a satellite project to Ref. 11. Thus, the study populations of Ref. 11 and the present study are essentially the same. Analogous to Ref. 11, patients were eligible for enrolment (i) if they were at least 65 years of age; (ii) if they were treated on the gerontopsychiatric ward of the Department of Psychiatry, Social Psychiatry and Psychotherapy of Hannover Medical School between April 2021 and February 2022; and (iii) if they or their legal guardian had provided written informed consent that patient data be used for research purposes. Hannover Medical School is a university hospital and tertiary care centre in northern Germany; the gerontopsychiatric ward is a 27‐bed facility specialised in the treatment and care of elderly psychiatric inpatients. In contrast to Ref. 11, patients taking drugs regularly and/or on an as‐needed basis (i.e., *pro re nata* (PRN) drugs) were eligible for enrolment (Ref. 11: only patients with regularly taken medication).

### Data acquisition

Ninety‐two individual patients were consecutively enrolled in our pilot study as a convenience sample between April 2021 and February 2022. Their medication charts were reviewed once weekly until discharge by an interdisciplinary expert team comprising specialists in psychiatry, internal medicine, geriatrics, and clinical pharmacology, yielding a total of 339 medication reviews. Since prescribing quality with regard to DPs was the focus of interest of the present study, all statistical analyses refer to *n* = 339 medication reviews as the denominator, unless stated otherwise.

### Definition and identification of ADPs and PIDPs

All drugs taken by the patients (both regularly taken drugs and PRN drugs) were analysed with the aid of the DP categorisation by Heck and colleagues.^5^ Medication charts of enrolled patients were screened for ADPs and PIDPs by an interdisciplinary expert team (MSW, SS, AG, HF, and SB: psychiatry; BK: internal medicine; AG: geriatric medicine; FK, DOS, and JH: clinical pharmacology).

### Outcome measures

The outcome measures were defined as the frequency and characteristics of PIDPs in elderly (i.e., ≥65 years of age) psychiatric inpatients, compared with the frequency of ADPs.

### Statistical analysis

We used descriptive statistical methods to summarise the data. Patient characteristics were not normally distributed and are therefore presented as medians with interquartile ranges (IQRs) for quantitative variables, and as absolute and relative frequencies for categorical variables. Differences between patients exposed to at least one PIDP (hereon termed patients with PIDPs) and patients not exposed to PIDPs (hereon termed patients without PIDPs) were evaluated with Pearson's chi‐squared tests for categorical variables (i.e., the variables ‘sex’, ‘presence of polypharmacy’, and ‘presence of ADPs’), whereas Mann–Whitney *U* tests were used for quantitative variables (i.e., the variables ‘age’ and ‘number of drugs taken’).^12^ *P*‐values (two‐sided) < 0.05 were considered statistically significant. Due to the exploratory nature of our study, no adjustments for multiple testing were conducted. Further details on the statistical methods are specified in Table 3. All statistical analyses were performed with IBM® SPSS® Statistics 28 (Armonk, New York, USA).

**Table 3:** Characteristics of the study population

Characteristic and category	Total	Patients without PIDPs	Patients with PIDPs	*P*‐value
	(*n* = 339)	(*n* = 193; 56.9%)	(*n* = 146; 43.1%)	
Median age (IQR)—years	73 (68–82)	72 (66–82)	73 (68–80)	0.335^†^
Female sex—% (no.)	64.6 (219)	68.9 (133)	58.9 (86)	0.056^‡^
Median number of drugs (IQR)	8 (6–11)	7 (4–10)	9 (7–12)	**<0.001** ^†^
Polypharmacy—% (no.)	81.7 (277)	73.1 (141)	93.2 (136)	**<0.001** ^‡^
Patients with ADPs—% (no.)	49.0 (166)	55.4 (107)	40.4 (59)	**0.006** ^‡^

^†^ Quantitative variables were tested for Gaussian distribution by utilisation of Shapiro–Wilk tests. Due to skewed distribution, differences between patients with and without PIDPs were analysed with two‐sided Mann–Whitney *U* tests for independent samples.^12^ Statistically significant *P*‐values (i.e., *P*‐values <0.05) are highlighted in bold.

^‡^ Pearson's chi‐squared tests were used to evaluate whether the presence of PIDPs was related to sex, presence of polypharmacy, or presence of ADPs. Statistically significant *P*‐values (i.e., *P*‐values <0.05) are highlighted in bold.

*Notes*: Patients with PIDPs were defined as patients exposed to at least one PIDP, while patients without PIDPs were defined as patients not exposed to PIDPs. Patients with ADPs were defined as patients who displayed at least one ADP. Polypharmacy was defined as the concomitant intake of ≥5 different drugs.

Abbreviation: ADP, appropriate duplicate prescription; IQR, interquartile range; PIDP, potentially inappropriate duplicate prescription.

## RESULTS

### Study population and medication chart reviews

Ninety‐two individual patients were enrolled in the study, for whom a total of 339 medication chart reviews were conducted (median number of medication chart reviews per patient: 3; IQR 2–5; range 1–20). The median age of the study population for whom medication chart reviews were performed (*n* = 339) was 73 years (IQR 68–82 years; range 65–94 years) and 64.6% (219/339) were female (Table 3). Patients' medications comprised a median of eight drugs (IQR 6–11 drugs; range 1–21 drugs). Disease characteristics of the study population have been reported elsewhere.^11^ In brief, the three most frequent psychiatric diagnoses in the study population (on the level of *n* = 92 individual patients) were dementia (39.1%; 36/92), depression (37.0%; 34/92), and schizophrenia/schizophreniform disorder (18.5%; 17/92). The most frequent somatic comorbidity was arterial hypertension (66.3%; 61/92), followed by atrial fibrillation (22.8%; 21/92) and coronary heart disease (16.3%; 15/92).

### PIDPs

One hundred and forty‐six of 339 patients for whom medication chart reviews were performed (43.1%) were exposed to at least one PIDP. As demonstrated in Table 3, there were no statistically significant differences in terms of age or sex between patients with and without PIDPs.

With respect to PIDP grades, 39.5% (134/339) of the study population were exposed to at least one grade‐1 PIDP, 5.0% (17/339) to at least one grade‐2 PIDP, and 1.5% (5/339) to at least one grade‐3 PIDP.

**Figure 1:**
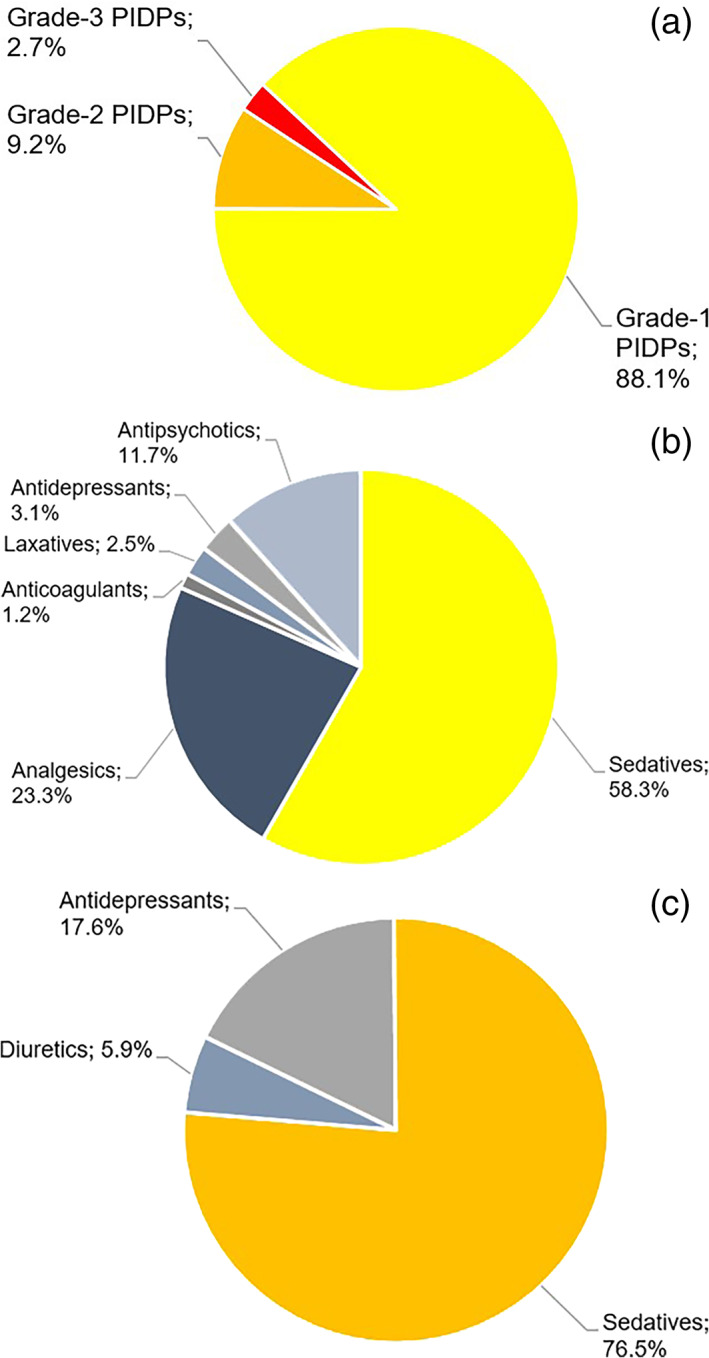
Subcategorisation of potentially inappropriate duplicate prescriptions (PIDPs) (*n* = 185) detected in the study population. (a) Distribution of PIDPs into grades 1–3. (b) Medication classes involved in grade‐1 PIDPs (*n* = 163). (c) Medication classes involved in grade‐2 PIDPs (*n* = 17). Grade‐3 PIDPs (*n* = 5) were exclusively elicited by analgesics (no pie chart shown).

Overall, 185 PIDPs were detected in the study population. The majority of PIDPs were of grade 1 (88.1%; 163/185) (Fig. 1a). Sedatives were most frequently responsible for grade‐1 PIDPs (58.3%; 95/163) (Fig. 1b), with lorazepam + pipamperone (49/95) being most commonly implicated (Table 4). Sedatives were also most frequently responsible for grade‐2 PIDPs (76.5%; 13/17) (Fig. 1c), with melperone + pipamperone (11/13) being the predominant combination (Table 4). Of note, all grade‐3 PIDPs were elicited by non‐opioid analgesics (100%; 5/5). Etoricoxib taken both regularly and on demand (thus exceeding the approved maximum daily dose of 120 mg), and metamizole taken both regularly and on demand (thus exceeding the approved maximum daily dose of 4000 mg) accounted for 40% (2/5) and 60% (3/5) of grade‐3 PIDPs, respectively (Table 4).

**Table 4:** Absolute and relative frequencies of potentially inappropriate duplicate prescriptions detected in the study population

PIDPs	*n*	%^†^
Grade‐1 PIDPs	163	100
Antidepressants	5	3.1
Opipramol + venlafaxine	1	0.6
Paroxetine + trazodone	1	0.6
Sertraline + trazodone	3	1.8
Antipsychotics	19	11.7
Amisulpride + aripiprazole	2	1.2
Amisulpride + clozapine	1	0.6
Amisulpride + olanzapine	3	1.8
Amisulpride + risperidone	1	0.6
Aripiprazole + olanzapine	1	0.6
Clozapine + risperidone	1	0.6
Haloperidol + olanzapine	2	1.2
Haloperidol + quetiapine	2	1.2
Olanzapine + risperidone	1	0.6
Quetiapine + risperidone	5	3.1
Sedatives	95	58.3
Alprazolam + zopiclone	2	1.2
Pipamperone + valerian	6	3.7
Chlorprothixene + lorazepam	5	3.1
Diazepam + pipamperone	5	3.1
Lavender oil + pipamperone	2	1.2
Lorazepam + melperone	3	1.8
Lorazepam + pipamperone	49	30.1
Lorazepam + promethazine	1	0.6
Lorazepam + zopiclone	1	0.6
Melperone + oxazepam	2	1.2
Oxazepam + pipamperone	5	3.1
Pipamperone + low‐dose quetiapine	5	3.1
Pipamperone + zolpidem	2	1.2
Pipamperone + zopiclone	7	4.3
Analgesics	38	23.3
Diclofenac + metamizole	6	3.7
Ibuprofen + metamizole	20	12.3
Ibuprofen + paracetamol	4	2.5
Metamizole + paracetamol	7	4.3
Paracetamol + tramadol	1	0.6
Anticoagulants	2	1.2
Edoxaban + tinzaparin	2	1.2
Laxatives	4	2.5
Lactulose + macrogol	4	2.5
Grade‐2 PIDPs	17	100
Antidepressants	3	17.6
Citalopram + sertraline	1	5.9
Duloxetine + venlafaxine	2	11.8
Sedatives	13	76.5
Alprazolam + oxazepam	1	5.9
Lorazepam + oxazepam	1	5.9
Melperone + pipamperone	11	64.7
Diuretics	1	5.9
Furosemide + torasemide	1	5.9
Grade‐3 PIDPs	5	100
Analgesics	5	100
Etoricoxib taken regularly and on demand (exceeding the approved maximum daily dose of 120 mg)	2	40
Metamizole taken regularly and on demand (exceeding the approved maximum daily dose of 4000 mg)	3	60

^†^ Percentages may not total 100 because of rounding.

Abbreviation: PIDP, potentially inappropriate duplicate prescription.

### ADPs

In nearly half of all patients for whom medication chart reviews were conducted (49.0%; 166/339), at least one ADP was present (hereon termed patients with ADPs) (Table 3). The proportion of patients with ADPs was significantly lower among patients with PIDPs than among patients without PIDPs (40.4% (59/146) vs 55.4% (107/193); *P* = 0.006).

### Polypharmacy

Polypharmacy (i.e., the concomitant intake of ≥5 different drugs^13^) was present in 81.7% (277/339) of the study population (Table 3). A significantly higher proportion of patients with PIDPs were affected by polypharmacy compared to patients without PIDPs (93.2% (136/146) vs 73.1% (141/193); *P* < 0.001). Accordingly, patients with PIDPs took significantly more drugs than patients without PIDPs (median nine drugs (IQR 7–12 drugs) vs seven drugs (IQR 4–10 drugs); *P* < 0.001).

## DISCUSSION

The present pilot study sought to investigate the frequency and characteristics of DPs in a cohort of elderly psychiatric inpatients treated in a university hospital in Germany. The analysis was based on weekly medication chart reviews conducted by an interdisciplinary expert team. We detected at least one PIDP in 43.1% of all reviewed medication charts. Of note, 39.5%, 5.0%, and 1.5% of the study population were exposed to at least one grade‐1, grade‐2, and grade‐3 PIDP, respectively. Sedatives accounted for the majority of grade‐1 and grade‐2 PIDPs, while non‐opioid analgesics were responsible for all grade‐3 PIDPs. Lorazepam + pipamperone and melperone + pipamperone represented the most frequent grade‐1 and grade‐2 PIDP combinations, respectively. Etoricoxib taken regularly + etoricoxib PRN and metamizole taken regularly + metamizole PRN constituted the grade‐3 PIDP combinations. Of interest, patients with PIDPs took significantly more drugs than patients without PIDPs, and—accordingly—were significantly more often affected by polypharmacy.

Almost half of the study population displayed ADPs. This is an important finding because previous studies did not sufficiently discriminate between beneficial and potentially harmful DPs. Instead, other authors regarded DPs exclusively as deleterious to patients' health,^14^, ^15^, ^16^, ^17^, ^18^ a notion that appears to be challenged by our study results.

When comparing our results with those of other studies we must stress that there is a plethora of different terminologies and definitions of DPs (Table 1; demonstrated by the use of quotation marks upon first appearance in the following text), which substantially limits comparability.

Zwietering *et al*. found a very low (between 0% and 1.9%) prevalence of ‘double medications’ in geriatric outpatients,^15^ suggesting that outpatients may be less affected by PIDPs than inpatients, and that psychiatric illness may pose a particular risk factor for the occurrence of PIDPs.

Kinoshita and colleagues reported an 8.8% prevalence of ‘duplicative medications’ among insurants of a corporate health insurance society.^16^ Of note, their study population was considerably younger than ours (mean age (± standard deviation) 33.4 (±17.5) years^16^ vs median age (IQR) 73 years (68–82 years)), which makes it very difficult to compare our study results with Kinoshita's. Furthermore, Kinoshita *et al*. analysed health insurance data,^16^ while we strictly focused on clinical data. Astonishingly, whereas antibiotics represented a common cause of duplicative medications in the Kinoshita *et al*. study,^16^ they were not implicated in any PIDPs in our study, a discrepancy that might be explained by the fact that all DPs of antibiotics in our study were classified as ADPs, while Kinoshita *et al*. did not distinguish between appropriate and inappropriate DPs.^16^

According to Heinze and co‐workers, 13–15% of the Austrian population taking antihypertensive, antilipidemic, or antidiabetic agents were affected by ‘double medications’.^14^ However, their applied methodology did not allow for a discrimination between appropriate and inappropriate double medications. Besides, detection of grade‐3 PIDPs was not possible with Heinze *et al*.'s definition of double medications.^14^

In a cross‐sectional study of potentially inappropriate prescribing in older patients admitted to a psychiatric hospital in The Netherlands, benzodiazepines, antipsychotics, and antidepressants most frequently accounted for ‘any regular duplicate drug class prescription’.^10^ The definition used by the authors (which was based on STOPP criteria) resembled our definition of grade‐2 PIDPs, albeit without differentiating between appropriate and potentially inappropriate DPs. Therefore, it remains elusive whether, for example, the (clinically indicated) co‐administration of two platelet aggregation inhibitors (e.g., low‐dose acetylsalicylic acid plus a P2Y_12_ receptor antagonist) subsequent to coronary stent implantation^19^ would have been assessed as a ‘regular duplicate drug class prescription’. This again demonstrates the advantage of our DP categorisation (which does differentiate between ADPs and PIDPs) compared to other DP definitions (Table 1).

Analgesics accounted for 23.3% of grade‐1 PIDPs in our study, with ibuprofen + metamizole being the predominant combination. Similarly, in a retrospective cohort study among multimorbid elderly outpatients, diclofenac + metamizole were most frequently responsible for ‘DPs’ equivalent to grade‐1 PIDPs.^18^ Grade‐2 PIDPs due to analgesics were not observed in our study; however, all grade‐3 PIDPs were elicited by analgesics. In the abovementioned study,^18^ two DPs equivalent to grade‐3 PIDPs were detected, both elicited by diclofenac + diclofenac. In our study, by contrast, etoricoxib + etoricoxib and metamizole + metamizole were responsible for grade‐3 PIDPs.

Kovac and colleagues investigated ‘dual use’ of analgesics and demonstrated a correlation between dual non‐steroidal anti‐inflammatory drug (NSAID) use and worse physical health‐related quality of life, albeit in a highly selected patient population (their study population consisted exclusively of NSAID users).^17^ According to the DP categorisation applied in our study,^5^ dual NSAID use as defined by Kovac *et al*.^17^ is equivalent to grade‐2 or grade‐3 PIDPs. Although grade‐2 PIDPs due to analgesics were not observed in our study, and although Kovac *et al*. analysed the effects of dual use in only one medication class (i.e., NSAIDs), their results suggest that PIDPs might in fact be harmful to patients.

A strength of our study is the large number of medication chart reviews which were conducted by an interdisciplinary expert team comprising specialists from psychiatry, internal medicine, geriatrics, and clinical pharmacology. We are convinced that utilisation of the DP categorisation by Heck *et al*.^5^ minimised subjective bias and abrogated the blending of clinically beneficial and detrimental DPs, which was a major limitation of previous works (Table 1). On the other hand, the relatively short duration of our pilot study, as well as its retrospective and monocentric design clearly represent limitations. Our study was carried out at a single university hospital; therefore, the results may not be generalisable to other settings in which elderly psychiatric patients are treated and cared for.

The most important limitation of our study is the lack of information about the number of cases in which PIDPs actually resulted in adverse events. Such an evaluation was not feasible due to the retrospective character of our study, which did not allow for a precise and unbiased ADR causality assessment. Future prospective investigations, however, should evaluate the clinical significance of PIDPs, for example, by systematically analysing PIDP‐associated ADRs immediately upon occurrence with established causality assessment tools.^20^, ^21^

Even though the clinical implications of PIDPs are not fully established to date, we recommend that physicians who treat elderly psychiatric patients pay special attention to PIDPs, especially PIDPs elicited by sedatives. Termination of PIDPs due to sedatives may not only reduce the risk of cognitive impairment and the risk of falls, but may also save healthcare expenditures caused by unnecessary drugs in an ageing society.

## AUTHOR CONTRIBUTIONS

MSW, SS, BK, and JH conceptualised the study. MSW, SS, FK, BK, and JH analysed the study data. BK provided statistical advice. BK and JH conducted the statistical analyses. BK devised the tables. JH created the figure. All authors contributed to the interpretation of the study results. MSW, SS, AG, and JH wrote the first draft of the manuscript. All authors commented on previous versions of the manuscript. All authors read and approved the final version of the manuscript. The corresponding author (JH) attests that all listed authors meet authorship criteria and that no persons meeting the criteria have been omitted.

## DISCLOSURE

The authors declare that there are no conflicts of interest. There are no funders to report for this submission.

## Data Availability

The data that support the findings of this study are available from the corresponding author upon reasonable request.
